# Application of the “hospital-school-home-community” integrated mental health service model for primary and secondary school students in Chongqing, China

**DOI:** 10.3389/fpsyt.2025.1554939

**Published:** 2025-06-27

**Authors:** Su Hong, Qi Zhang, Yifeng Wei, Ming Ai, Wo Wang, Jian-mei Chen, Xiao-ming Xu, Xinmin Li, Andrew James Greenshaw, Li Kuang

**Affiliations:** ^1^ Psychiatric Center, First Affiliated Hospital of Chongqing Medical University, Chongqing, China; ^2^ Department of Psychiatry, University of Alberta, Edmonton, AB, Canada; ^3^ Mental Health Center, University-Town Hospital of Chongqing Medical University, Chongqing, China

**Keywords:** adolescents, mental health, mental health literacy, intervention, healthcare

## Abstract

**Background:**

Childhood and adolescence are critical periods for mental health development, with a significant risk for mental disorders. The Chinese government has prioritized student health and well-being, encouraging policies to support youth mental health. However, challenges remain due to high technical demands and inter-departmental coordination.

**Methods:**

The “pathway through care” model connects hospitals, schools, communities, and families to collaboratively promote adolescent mental health. The model employs general screening, interviews, and intervention strategies at various levels, including mental health promotion and training for students, educators, and parents. The model’s operation is led by a tertiary care team in collaboration with school districts.

**Results:**

Since the implementation of the model in Chongqing, a large-scale screening of students has been conducted, with over 330,000 students screened and a significant number receiving face-to-face interviews with psychiatrists/psychologists. The model has facilitated the early identification of students at risk, appropriate referrals for care, and ongoing support within the school setting. It has also engaged parents and the wider community in addressing youth mental health needs.

**Conclusions:**

The “Hospital-School-Home-Community” integrated mental health service model has demonstrated success in improving child and youth mental health within the school setting in Chongqing. It has effectively linked education and health systems to address a full continuum of care.

## Background

Childhood and adolescence are critical periods when many of the substantive and persistent mental disorders manifest, including major depressive disorder, panic disorder, bipolar disorder, substance abuse, eating disorders, and schizophrenia (1). Neuropsychiatric disorders comprise the largest single category of medical disability in young people globally (2–4). Unrecognized and untreated mental disorders can lead to a variety of negative long- and short-term outcomes, such as poor educational and vocational achievement, problematic social and personal functioning, and reduced life expectancy due to associated medical conditions and suicide (5–7).

Unfortunately, in the wake of global COVID-19 epidemic, students’ mental health problems have become a more salient social issue that demands heighted attention (8). A meta-analysis of 15 studies involving 24,055 participants revealed a prevalence of non-suicidal self-injury (NSSI) related to mental health problems in adolescents during the pandemic, reaching 32.4% (9), up from 19.5% before the pandemic in children and youth (10). Additionally, the rates of chronic stress, anxiety, and depression increased in children and youth (11). A report released by the United Nations International Children’s Emergency Fund (UNICEF) warns that governments need to pay attention to the worsening mental state of children and young people around the world. In China, with the rapid economic and social development, alongside an evolving environment, the prevalence of mental illness among children and adolescents is increasing. A national psychiatric epidemiological survey conducted among children and adolescents indicates that the weighted prevalence of any mental disorder is 17.5% (95% CI: 17.2-18.0) the highest prevalence ever reported in China since 1990s (12–15). Sorted by DSM-IV disorder categories, the prevalence of attention-deficit and disruptive behavior disorders was 10.2% (95% CI: 10.0-10.4); anxiety disorders, 4.7% (95% CI: 4.6-5.0); and depressive disorders 3.0 (95% CI: 2.8-3.1) (16). In Chongqing, regional screening data revealed that self-harm prevalence among 48,117 preadolescents was 13.6% (n=6561) (17). Addressing these challenges is imperative for public mental health service providers, policymakers and the society at large.

Promoting student health and well-being has long been a priority for the Chinese central government (18–20), which encourages provincial and local governments to develop polices to support child and youth mental health. For instance, Chongqing Municipality, one of the largest municipalities in China has issued the Notice of the Chongqing Municipal Education Commission on *Strengthening and Improving Mental Health Education in Primary and Secondary Schools in the New Era* as well as the *Notice of the Chongqing Municipal Education Commission on Establishing and Improving the Working Mechanisms for Psychological Crisis Intervention for Primary and Secondary Students*, in alignment with national policies. Although the government recognizes the importance and urgency of this endeavor, the high technical requirements for psychological and mental health services and the challenges associated with inter-departmental coordination have prevented the development of a comprehensive and executable student psychological and mental health service model at the national or provincial level.

Evidence suggests that preventing and intervening mental disorders among students requires cooperation among hospitals, schools, and families to develop a pathway through care model for coherent and consistent support of students in need of care (21). The School Mental Health International Leadership Exchange (SMHILE) has identified five critical themes for advancing global school mental health: cross-sector collaboration in building systems of care, meaningful youth and family engagement, workforce development and mental health literacy, implementation of evidence-based practices, and ongoing monitoring and quality assurance (22). Globally, partnerships between schools and other youth-serving systems are enhancing more comprehensive school-based mental health services, advocating for and promoting collaborations among education, health, and the community. In China, following approval by the Central Establishment Office in 2021, the National Center for Mental Health was established to facilitate interdisciplinary collaboration among relevant departments and agencies. Provincial government agencies are mandated to establish a mental health leadership groups or multi-departmental coordination mechanisms at provincial, municipal, and county levels. Local governments at all levels are instructed to establish technical management bodies for mental health, focusing on training, supervising, organization, coordination, and the specific implementation of prevention, treatment, rehabilitation, and health education (23).

In China, addressing student mental health involves comprehensive societal efforts, necessitating collaboration among education, healthcare, insurance, justice departments and other community organizations to fulfill their respective responsibilities, with broader societal participation (24). Significant progress has been achieved in the development of student mental health services, including the establishment of mental health counseling centers for minors at the prefectural and county levels, and the provision of mental health care services for children in over 1,600 maternal and child health care institutions nationwide. The government has also recruited mental health professionals, to develop child psychological screening forms for primary care child health care providers enabling early identification of warning signs of mental disorders. Moreover, systems for screening and intervening with children with autism has been established and enhanced. The government supports local initiatives aimed at preventing and treating common mental disorders and promoting mental health of children and adolescents. The Ministry of Education of China, in collaboration with the National Health Commission, the Chinese Academy of Sciences and education experts has jointly developed mental health service packages and peer support toolkits that are tailored to the characteristics and needs of Chinese adolescents (20). The Ministry of Education has guided local education authorities in compiling and distributing the provincial “Guidance Manual for Children’s and Family Mental Health Education”, and carried out family education guidance and psychological care for children in difficult circumstances in rural areas (25). However, significant challenges remain. Some local schools lack proactive coordination in the implementation of mental health responsibilities due to the misunderstandings or insufficient understanding about mental health, compounding by overwhelming daily working schedules. In particular, stakeholders - including students, parents, teachers, and education administrators - have insufficient understanding of mental health, as well as widespread stigmatizing attitudes, hindering the formation of a unified approach and momentum to address child and youth mental health.

These barriers may be mitigated by evidence-based mental health literacy (MHL) approaches. MHL has been defined as the knowledge and skills necessary to foster mental health (26) as a functional literacy and is associated with improved mental health outcomes. It has evolved as an empowerment tool that address four interrelated domains: understanding how to obtain and maintain positive mental health; understanding mental disorders and their treatments; decreasing stigma related to mental disorders; and, enhancing help-seeking (27). MHL has been recognized as an evidence-based strategy in promoting the youth’s mental health, early identifying youth at risk, and therefore potentially benefiting both individual and public mental health (27, 28). However, interventions targeting adolescents and aiming to improve MHL and promote behavior changes are lacking in China (29).

In order to support the prevention and treatment of common mental disorders and the promotion of mental health among children and adolescents, the Chongqing district-level government and Chongqing Medical University have jointly integrated social resources to have developed mental health service packages and peer support toolkits that meet the characteristics of Chinese adolescents. This service is implemented under the concept of linked service among schools, families and the health care, advocated and evidenced by solid research evidence (22, 30). Since 2020, this mental and psychological service has been provided to children and youth from primary schools (usually 7–13 years old), primary and junior secondary schools (7–16 years old), junior secondary schools (13–16 years old), high schools (16–18 years old), and secondary vocational schools (16–18 years old).

This study aims to address the following research question: How can an integrated mental health service model, tailored to China’s socio-cultural context, improve early identification, intervention efficacy, and stakeholder engagement for adolescents in Chongqing? The objectives are: (1) to design a culturally adaptive “Hospital-School-Home-Community” model addressing gaps in existing systems (e.g., fragmented referrals, low mental health literacy); (2) to evaluate the model’s feasibility through large-scale screening and stakeholder training outcomes. (3) to provide actionable insights for scaling the model in regions with diverse healthcare resources. Grounded in the social-ecological model, this framework emphasizes multi-level interactions between individuals, institutions, and policies, offering a theory-driven approach to systemic mental health challenges.

## Chongqing pathway through care service model

We hereby describe the Chongqing Pathway through Care Service model that has been developed and implemented in Chongqing Municipality, which hosts a population of more than 30 million, making it one of the largest municipalities in China and globally. This model aims to improve child and youth mental health within the school setting. It is inspired by the Canadian model (21) that applies the mental health literacy approach within and beyond the school setting to link schools, the health system and the community together to promote student mental health. Building on this foundation, we further consider the social and cultural context of Chongqing, recognizing the importance to early identify students at risk of mental illness through universal screening. These students can be immediately supported within the school and further referred to the healthcare system if needed. In parallel with early identification, triage and intervention, mental health literacy is implemented among students, parents/families, teachers and other related stakeholders, which enhances early identification, prevention, and promotion efforts. Together we create a green pathway through a care service model in which we engage a cadre of professionals and related stakeholders in education and health to provide services at various points along the pathway.

The design of this model is grounded in the Social-Ecological Model, systematically addressing adolescent mental health needs through multi-level intervention strategies. At the individual level, universal screening tools (e.g., CDI, PHQ-9 in **Table 1**) enable early risk identification, while mental health literacy (MHL) curricula (e.g., “Mental Health Curriculum” in **Table 2**) enhance students’ self-regulation and help-seeking efficacy. At the interpersonal level, parent-school co-education programs (e.g., “Parent Courses” in **Table 2**) strengthen family communication skills and crisis management, aligning with Confucian cultural values that position families as central support units. At the community level, the “Psychology Cloud Platform” bridges schools, hospitals, and community resources (e.g., “Gatekeeper Training” in **Table 2**), forming a cross-sector collaboration network to reduce service fragmentation. At the policy level, institutional mandates from central and local governments (e.g., *Notice on Strengthening Mental Health Education in the New Era*) ensure sustainable resource allocation and policy implementation. This multi-tiered design not only reflects the Social-Ecological Model’s core principle of “individual-environment interaction” but also enhances the model’s effectiveness in East Asian contexts through culturally adaptive strategies (e.g., family-centric interventions). It thus offers a theory-driven framework for similar regions seeking to balance systemic rigor with cultural relevance. The pathway model is operated by the Psychiatry Department of the First Affiliated Hospital at the Chongqing Medical University in collaboration with school districts in the region. The operation mechanism is the first of its kind, where a tertiary care team including psychiatrists, psychotherapists, and social workers, leads the community-based child and youth mental health care, encompassing promotion, prevention, and care between the education and healthcare system.

**Table 1:** Scales used in student mental health screening.

Student level	Instruments
Primary students	General Information Questionnaire (Primary School Version)
	Children Depression Inventory (CDI)
	General Anxiety Disorder (GAD-7)
Junior secondary school students	General Information Questionnaire (Secondary School Version)
	Adolescent Self-Rating Life Events Checklist (ASLEC)
	Internet Addiction Test (IAT)
	General Anxiety Disorder (GAD-7)
	Suicide Self-Injury Screening (Secondary School Version)
	Patient Health Questionnaire-9 (PHQ-9)
	Patient Health Questionnaire-15 (PHQ-15)
High school students	General Information Questionnaire (High School Version)
	Symptom Checklist-90 (SCL-90)
	Quality of Life
	Internet Addiction Test (IAT)
	Barratt Impulsiveness Scale (BIS-11)
	Suicide Self-Injury Screening (High School Version)
	Aggression Questionnaire
	Patient Health Questionnaire-9 (PHQ-9)
	General Anxiety Disorder (GAD-7)
	Patient Health Questionnaire-15 (PHQ-15)

**Table 2:** Examples of school mental health promotion programs corresponding to the comprehensive school mental health model.

	Program	Target audience	Project description	Partners
Mental health curriculum	Mental health curriculum for implementation in primary, secondary and high schools.	Students in Grade 4 and above	Topics include: positive mental health, emotion self-adjust, Interpersonal communication, internet use	Chongqing Municipal Education Commission, Government Smart Education Cloud Media Platform, Chongqing Medical University, Southwest University, Army Medical University
Parent Courses	Home-school co-education curriculum	Parents	Focus on parent-child communication and symptom management and family interventions. Topics include adolescent communication, sexual development, parent-child companionship	
Psychological Skills Course for Teachers	Psychologically Theoretical Foundations to identify early manifestations of self-injury, suicide, and mental problems	Educators, student services providers, school, school administrators, community health providers	Early identification and crisis intervention of mental and psychological problems	
Gatekeeper training	Understanding common mental and psychological problems and crisis intervention	Educators, student services providers, school, school administrators, community health providers	Training in topics including symptoms, causes, identification and treatment of depression, prevention, and screening for suicide risk	Chongqing Municipal Education Commission, Chongqing Medical University

Based on the needs of youth and their families, the goals of this service model are: 1) to enhance formal linkages between healthcare providers and schools; 2) to promote appropriate and timely access to mental health care through early identification, triage and evidence-supported mental health interventions; 3) to promote mental health and reduce stigma by enhancing the mental health literacy of student, educators and parents; and 4) to involve parents and the wider community in addressing the mental health needs of youth.

## Early identification, triage and intervention

Screening for mental health problems in schools has been controversial due to factors such as social, cultural, linguistic challenges (31). However, universal screening for mental health problems is necessary to reach youth who otherwise would fall through cracks, especially in the Chongqing social and cultural context. In Chongqing, schools have already built long-term collaborations and trust with the health system, including the First Affiliated Hospital of Chongqing Medical University, supported by the government policy and funding. Additionally, the Chinese tradition that families rely heavily on the education system to support not only academic performance of their children but also their health, including mental health. A number of systematic reviews and meta-analysis summarized the prevalence of depression among Chinese youth ranging from 19.85 to 28.6% (32, 33), a much greater rate compared to the worldwide prevalence of 6.2% among youth (34, 35). Suicide, which can result from the interaction of mental disorders such as depression, was the third leading cause of death among adolescents aged 12–18 years in China (36), highlighting the urgent need to address these challenges effectively and efficiently.

In order to early identify students at risk of mental disorders, the project team developed screening programs for mental health problems such as depression, anxiety, self-harm and suicidal behaviors among students in primary schools, junior secondary schools, and high schools, respectively (**Table 1**). The scales used in the screening were age appropriate and all tested in the Chinese adolescent population and demonstrated good reliability and validity (37–44). Recognizing the logistic limitations of the in-person screening, typically hosted by teachers and school staff who have to receive training, and the unavailability of school health professionals and primary care providers in many schools, we have created and utilized the online system of “Chongqing Medical University Psychology Cloud Service Platform”. An online assessment is conducted in September every academic year to establish and update students’ psychological files in real time. Completion rates for online psychological assessments exceed 95%, with invalid responses (e.g., inconsistent answers) excluded through automated validity checks. Teachers followed up with students who missed screenings. The cloud platform uses algorithm thresholds (e.g., PHQ-9≥15 for depression, GAD-7≥15 for anxiety) validated in Chinese adolescents. Based on the results of psychological testing, the system automatically identifies students with possible depression, anxiety, somatic symptoms, and self-harm/suicide behaviors. Students exceeding thresholds are flagged for interviews. Our team members who are psychiatrists from the hospital monitor and analyze the screening results. These students are then interviewed face-to-face by psychologists and psychiatrists with the Structured Clinical Interview for DSM-5 Disorders (SCID-5) for further assessment to determine their psychological risk level. After the interview, senior psychiatrists from the hospital quality control the interview results. Then, the medical team reports the results of the student’s mental health assessment to the District Board of Education, which advises the vice principal in charge of student mental health education at each school on how to handle the student’s mental health status. The vice principal then relays the information to the corresponding class teacher. The results of the students’ psychological assessments are strictly confidential, and only two administrators in charge of the medical team and the District Board of Education have access to all students’ psychological files. This process alleviates the usual confidentiality concerns associated with screening for mental health problems in schools.

In order to provide rapid and effective mental health services to students in need of psychiatric interventions (e.g., medication, hospitalization) or evidence-based psychological therapies (e.g., CBT, DBT) delivered by licensed professionals, the government has signed a joint agreement with the First Affiliated Hospital of Chongqing Medical University. This agreement facilitates direct access to medical treatment for students identified through screening as needing medical treatment and for those who experience acute psychological crises, ensuring that students can receive mental health services on the same day. Even during the COVID-19 pandemic, students with severe mental health problems and psychological crises were ensured referral to a medical facility for mental health services within 48 hours.

**Figure 1:**
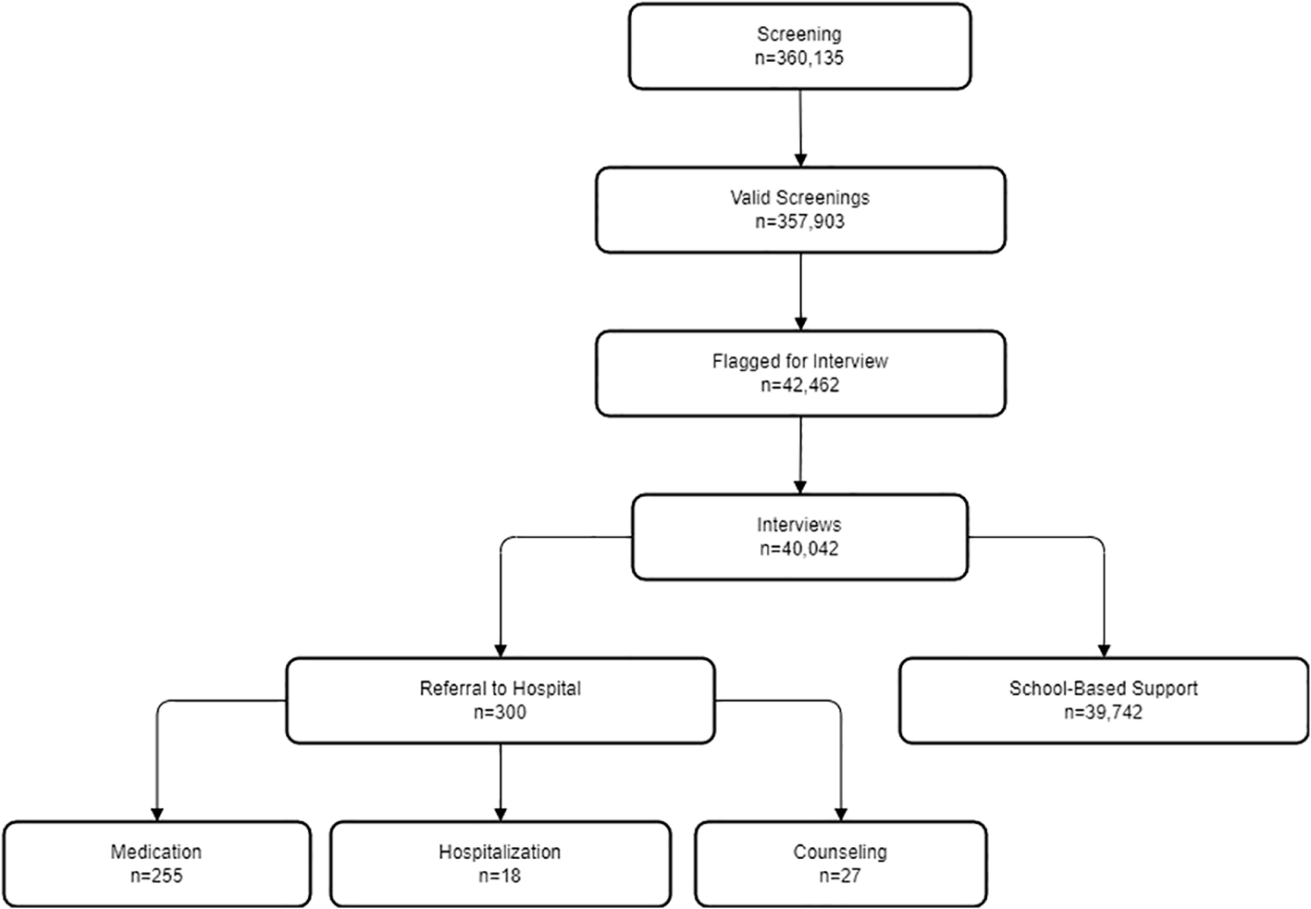
Flowchart of the study.

Since September 2020, more than 360,000 students have been screened, including 184,526 elementary school students, 103,047 junior high school students, and 72,563 high school students. A total of 40,042 students received face-to-face interviews with psychiatrists/psychologists. More than 300 students who were screened for possible mental problems such as high risk of suicide, major depressive disorder, anxiety disorder, and attention deficit hyperactive disorder were referred to the First Affiliated Hospital of Chongqing Medical University for psychiatric outpatient/inpatient treatment or psychotherapy. Among the students referred for psychiatric care, 85% underwent psychiatric evaluation and were prescribed medication based on clinical need, consistent with guidelines for moderate-to-severe cases (e.g., SSRIs for depression, antipsychotics for psychosis). Additionally, 6% of the students exhibited psychotic symptoms, self-harm tendencies, and suicidal ideation and behaviors, prompting immediate hospitalization recommendations. Furthermore, 9% of the students were advised to enhance their mental health education, modify family communication strategies, engage in tailored psychological counseling, and implement regular follow-up sessions. Of the students referred for psychiatric treatment, 12% successfully resumed regular schooling after completing treatment, reflecting a phased reintegration process supported by school-based follow-up. Meanwhile, 51% of the referred students were temporarily suspended due to severe symptoms (e.g., acute suicidality, psychosis) requiring intensive care. Suspensions were mandated by school policies to ensure student safety and well-being during treatment, with their respective class teacher or psychology teacher conducting monthly follow-up visits. The outcomes of these visits were subsequently uploaded to the “Chongqing Medical University Psychology Cloud Platform” for tracking. Unfortunately, 37% of the students could not be accounted for as they had transferred to another school, dropped out, or declined to participate in the school’s survey (**Figure 1**).

## Promotion, prevention and intervention

To improve the mental health of student and enhance the capacity of teachers, students service providers, and parents/families to address students’ psychological problems, the project team has implemented hierarchical interventions through a combination of online course resources and face-to-face mental health literacy professional training. We adopted the mental health literacy (MHL) approach (27) as the intervention strategy to build a shared language among all related stakeholders, ensuring consistent attention to student mental health in the education, community/families and health care settings. Kutcher et al. (27) defined MHL as the knowledge about mental health and mental illness, capacity to achieve and sustain good mental health, help-seeking efficacy, and strategies to fight stigma towards mental illness (27). This approach has been extensively applied and studied to improve knowledge, reduce stigma, enhance help-seeking behaviors and improve the quality of referral between the systems (45–47). We developed the following MHL resources to respond to the needs of Chinese youth and related stakeholders. While formal MHL quantification was not systematically implemented across all participants due to scalability challenges, we embedded knowledge checks within online courses. For instance, teachers scored 78.4 ± 12.3 (out of 100) on post-course quizzes assessing crisis intervention principles, compared to 41.2 ± 18.6 in pre-course baselines (*P*<0.001).

*Online MHL resources*: The project team has produced targeted online resources and courses to provide MHL education for students, parents, and teachers including 11 topics for students, 38 topics for parents, and 6 topics for teachers that meet the mental health needs of these populations (**Table 2**). Topics for students focus on understanding how to achieve and maintain good mental health and self-regulation skills. For parents, the focus is on parent-child communication and symptom management and family intervention (e.g., How parents can help their children build self-confidence; what parents should do if their children are bullied; how parents deal with common emotional problems in adolescents). For teachers, the focus is on developing competencies for early identification and crisis intervention of mental and psychological problems. Examples of the teacher training resources include: Crisis Development- Student Psychological Crisis Management; Identification and response to common developmental and behavioral problems in children; Home-school collaboration makes dreams come true; Stress regulation and emotion management; Improving the psychological quality of students; Coping with problems in adolescent and children) (**Table 2**). The course materials are pre-recorded by the project team and Chongqing psychiatric professionals, and uploaded by the official online platform of the Chongqing Municipal Education Commission, garnering more than one million views. Stakeholder engagement metrics demonstrated active MHL adoption: 84% of teachers self-reported that they incorporated MHL concepts into parent-teacher meetings; student-initiated counseling requests rose from 5.2 to 11.7 per 1,000 student post intervention. Additionally, at the beginning and end of each academic year, the project team organizes two online live broadcasts by Chongqing psychiatric experts, who share the preparation tips and strategies that students, parents and teachers may take before the start of the academic year and the exam. Audiences can interact with the experts during these live broadcasts.

*Face-to-face training*: Psychological and MHL teacher training for both full-time and part-time staff is carried out twice a year by mental health professionals. This training is led by psychiatrists, psychotherapists, and counselors from the hospital, all of whom are all members of the national or international psychological rescue teams with experience in crisis intervention. The training course includes 8 hours of theoretical training and 8 hours of psychological intervention method and case drills. Theoretical training covers classification of psychological crisis, stress response, crisis intervention process, and emergency management model. Before the case drill and supervision session, the trainers collected the crisis cases experienced by the school psychology teachers, divided them into groups to play different roles and recreate the crisis scenes. Through this immersive experience, the trainees can discuss crisis management plans together, thereby increasing the crisis management experience.

## Longitudinal outcomes of symptom reduction

The sustained implementation of the “Hospital-School-Home-Community” model from September 2020 to October 2024 demonstrated significant improvements in student mental health outcomes. Longitudinal data collected through annual screenings revealed marked reductions in self-reported depressive and anxiety symptoms across all educational levels. Among elementary school students, the prevalence of self-reported depressive symptom declining from 17.9% to 14.0%. For junior high school students, depressive symptom drops from 5.3% to 4.2% and anxiety symptom from 4.8% to 3.8%. High school students exhibited the most substantial improvement in depressive symptoms, with a decline from 4.5% to 3.1%, alongside a reduction in anxiety symptoms (4.7% to 3.8%). These outcomes underscore the model’s effectiveness in mitigating mental health burdens through its multi-tiered approach, which integrates universal screening, stakeholder training, and timely psychiatric interventions. The observed reductions align with the model’s design goals of early identification, stigma reduction, and cross-system collaboration, highlighting its potential as a replicable framework for similar contexts.

## Discussions

This “Hospital-School-Home-Community” integrated mental health service model for primary and secondary school students is developed based on a well-conceived, evidence-based cohesive framework (27). This model addresses a variety of mental health needs from mental health promotion to case identification, triage, and referral; and from ongoing support within the school setting to the engagement of parents and the wider community. The model is consistent with the national and local realities of Chinese health and education systems, and encourages collaboration and sharing of information among organizations, agencies, and institutions across all sectors serving young people.

There have been calls for multi-disciplinary integrated mental health services for youth (48) to alleviate the challenges of lengthy, complicated journey for youth to reach appropriate mental health services. Successful examples include the ACCESS Open Mind (49) in Canada and the Headspace in Australia (http://www.headspace.org.au). However, a recent systematic review (50) on pathways to mental health services indicate that current pathway models in addressing youth mental health are mostly illness-based model with focuses on how to support young people to access to mental health care for treatment, usually with very few integrations of the promotion and prevention components and inadequate engagement of schools. Assessment of recent evidence indicates that provision of increased levels of mental health treatment may not have reduced prevalence of mental disorders (51). The review authors hypothesized that lack of sufficient and effective implementation of promotion and prevention activities in both health and education systems may underlie this observation. Schools are well-positioned to address child and youth mental health needs, as they are contexts where most children and youth spend their waking lives. Our model strives to bridge this significant gap by linking education and health systems to address a full continuum of care, backed up by the government policies and the buy-in among all stakeholders serving child and youth mental health.

While inspired by Canada’s “Pathway through Care” model (21), the Chongqing model demonstrates distinct advantages in policy integration, cultural adaptation, and implementation strategies. Unlike Canada’s decentralized framework reliant on NGO and provincial partnerships, the Chongqing model is driven by centralized government mandates (e.g., *Notice on Strengthening Mental Health Education in Primary and Secondary Schools in the New Era*), institutionalizing collaboration between education and healthcare systems to ensure stable resource allocation and policy enforcement. For screening and intervention, Chongqing innovatively combines online platforms (e.g., PHQ-9, GAD-7 scales via the Psychology Cloud Platform) with face-to-face interviews, addressing both cultural stigmatization (e.g., self-reporting bias) and logistical challenges of traditional screening. Furthermore, diverging from Western peer- or community-centric approaches (e.g., Australia’s Headspace), Chongqing’s model embeds Confucian cultural values by prioritizing family-centered interventions, such as 38 parental training modules on communication skills and crisis management. These features-policy coherence, hybrid screening mechanisms, and culturally tailored family engagement-position the Chongqing model as a replicable framework for low- and middle-income regions seeking to balance scalability with cultural sensitivity.

This pathway through care service model has been implemented in Chongqing for a number of years with success, evidenced by the number of students, parents and teachers involved and the endorsement of schools that have taken this model and longitudinal reductions in self-reported symptoms. Students with potential mental disorders have been appropriately identified, referred to health systems, provided with proper treatments and seamlessly returned to their schools – fully supported on their path to recovery. Yet, more advanced research and evaluation of this model are warranted to investigate the impact of the model, including the effectiveness of the MHL training among various stakeholders; and the quality of the screening and the effectiveness of treatment for students at risk of mental illness. In particular, the implementation and evaluation of the model components may be operated in accordance with the Reach, Effectiveness, Adoption, Implementation, and Maintenance (RE-AIM) Framework (52), which guides the implementation of evidence-based programs into real-world practices. As such, future research may explore the number/representativeness of schools and students who are willing to participate in the initiative and schools/institutions who are willing to sustain and continue the model implementation in the long run; the quality of the model protocol; and the effectiveness of the embedded interventions among students, educators and families alike. Once further validated, the Chongqing pathway through care model has great potential to be standardized and adopted by other jurisdictions in China and beyond.

The findings of this study hold significant implications for both policymakers and practitioners. For policymakers, the model demonstrates the feasibility of leveraging cloud-based platforms to bridge urban-rural disparities in mental health resource access, while its family-centric design—rooted in Confucian cultural—offers a replicable blueprint for other East Asian societies seeking to balance systemic scalability with cultural sensitivity. The government-led coordination between education and healthcare sectors, supported by municipal policies, underscores the importance of institutional mandates in sustaining cross-sector collaboration. For practitioners, the hierarchical framework (universal screening to tiered interventions) provides a pragmatic approach to balancing resource efficiency with individualized care, particularly in resource-constrained settings. To maximize impact, future initiatives should prioritize rural adaptations (e.g., e-mental health partnerships) and foster public-private collaborations to ensure equitable service delivery. These efforts, coupled with ongoing monitoring via the Psychology Cloud Platform, can drive long-term improvements in youth mental health outcomes across diverse socioeconomic contexts.

Although direct measures of MHL improvement remains a study limitation, three lines of evidence suggest literacy gains: The 58% reduction in stigma-related refusal for psychiatric referrals (2020: 34% vs. 2024: 14.3%) aligns with MHL’s destigmatization goals (27). Increased help-seeking from rural students reflects improved mental health service literacy. The integration of our MHL curriculum into Chongqing’s 2025 Mental Health Education Guidelines validates its perceived efficacy by policymakers.

This study has several limitations. First, 37% of the referred students were lost to follow-up, primarily due to school transfers, refusal to continue participation, or inability to contact. While anonymized data remain archived for potential future linkage, attrition may bias long-term outcome assessments. Second, reliance on online self-report tools (e.g., PHQ-9, GAD-7) introduces risks of underreporting stigmatized behaviors (e.g., self-harm) and technical barriers in rural areas with limited internet access. To mitigate this, future iterations will integrate teacher/parent observational checklists. Third, while the model demonstrates short-term efficacy, long-term sustainability remains untested. Fourth, the absence of standardized MHL metrics precludes definitive conclusions about literacy improvement, though behavioral proxies and policy uptake suggest positive trends. Future iterations will integrate validated MHL scales across all tiers. Finally, urban schools were overrepresented (75% of screened students), limiting generalizability to rural populations.

## Ethics statement

The study was approved by the Research Ethics Committee of the First Affiliated Hospital of Chongqing Medical University. All the participants recruited into the study were fully informed of the details of the study and agreed to participate. Informed consent followed a tiered protocol: parents/guardians of students under 18 provided written consent via school-distributed forms, available in both digital and paper formats. Minors aged 9–17 provided verbal assent after simplified explanations of study goals and confidentiality measures. The written informed consent was obtained from the participants ≧18 yrs, and permission from parents or legal guardians and assent from minors before the study. All data were anonymized and stored on encrypted servers hosted by Chongqing Medical University. Only two authorized administrators could access identifiers for urgent referrals. Annual audits ensured compliance with China’s Personal Information Protection Law (PIPL).
